# Supplementary material for: Coordinated Diel Gene Expression of Cyanobacteria and Their Microbiome
Source: Microorganisms. 2021 Aug 5;9(8):1670. doi: 10.3390/microorganisms9081670 (PMC8398468; doi:10.3390/microorganisms9081670)
Supplement: Supplementary file 1 [file microorganisms-09-01670-s001.zip › microorganisms-1297132-supplementary.pdf]

Coordinated diel gene expression of cyanobacteria and their microbiome

Kai Wang, Xiaozhen Mou*

Department of Biological Sciences, Kent State University, Kent, Ohio, United States

*Corresponding author: Xiaozhen Mou: xmou@kent.edu.

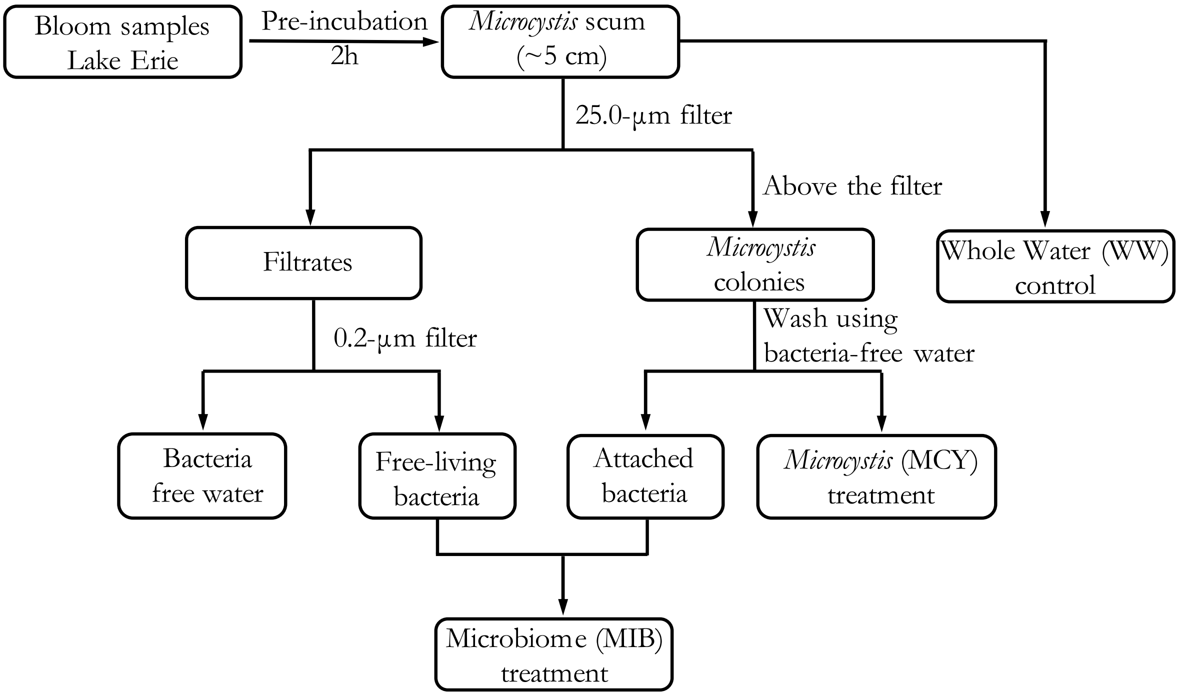

Figure S1 The diagram of microcosm experiment design.

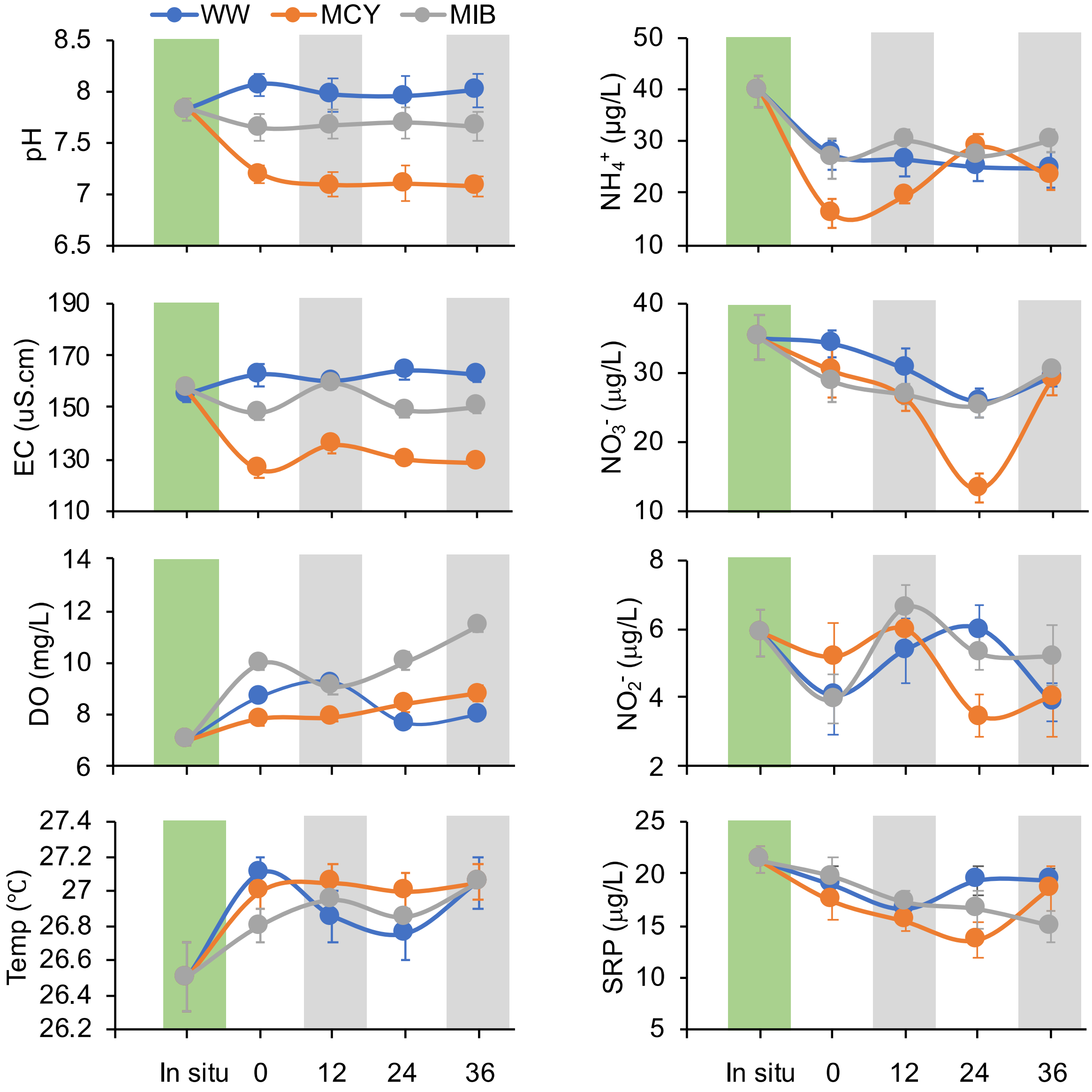

Figure S2. Measured physicochemical variables of Lake Erie and microcosm samples. EC: electronic conductivity; DO: dissolved oxygen; Temp: temperature; NH_4^+: ammonium; NO_3^-: nitrate; NO_2^-: nitrite; SRP: soluble reactive phosphorus. Green bars indicate preincubation samples, and gray bars indicate dark condition of the microcosm samples.

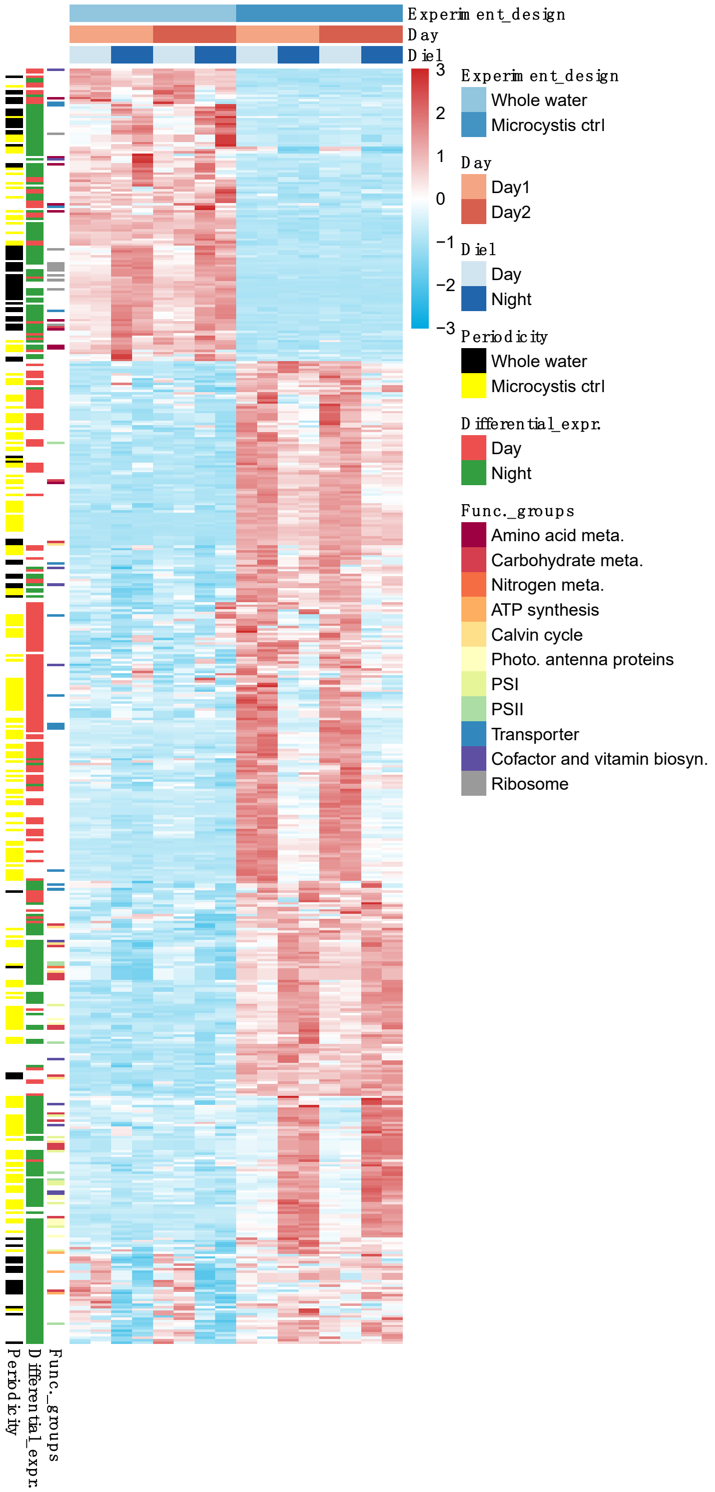

Figure S3 The expression pattern of Microcystis genes between the Microcystis (MCY) and whole water (WW) samples.

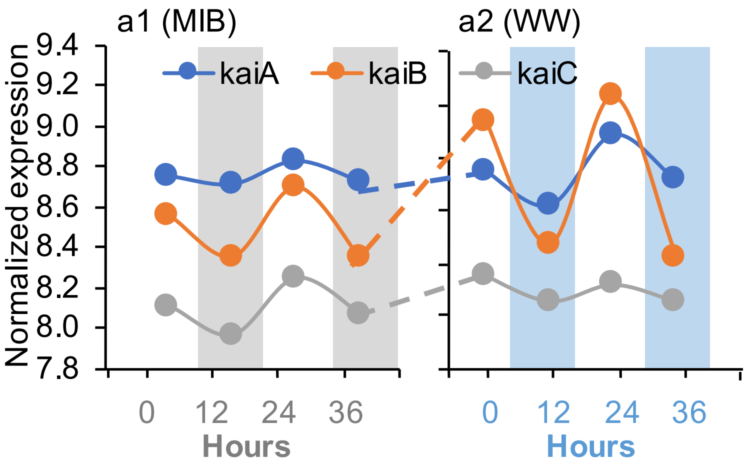

Figure S4. Averages of normalized Microcystis gene expression values. Calculations were based on varianceStabilizingTransformation in DeSeq2 related to circadian clock genes.

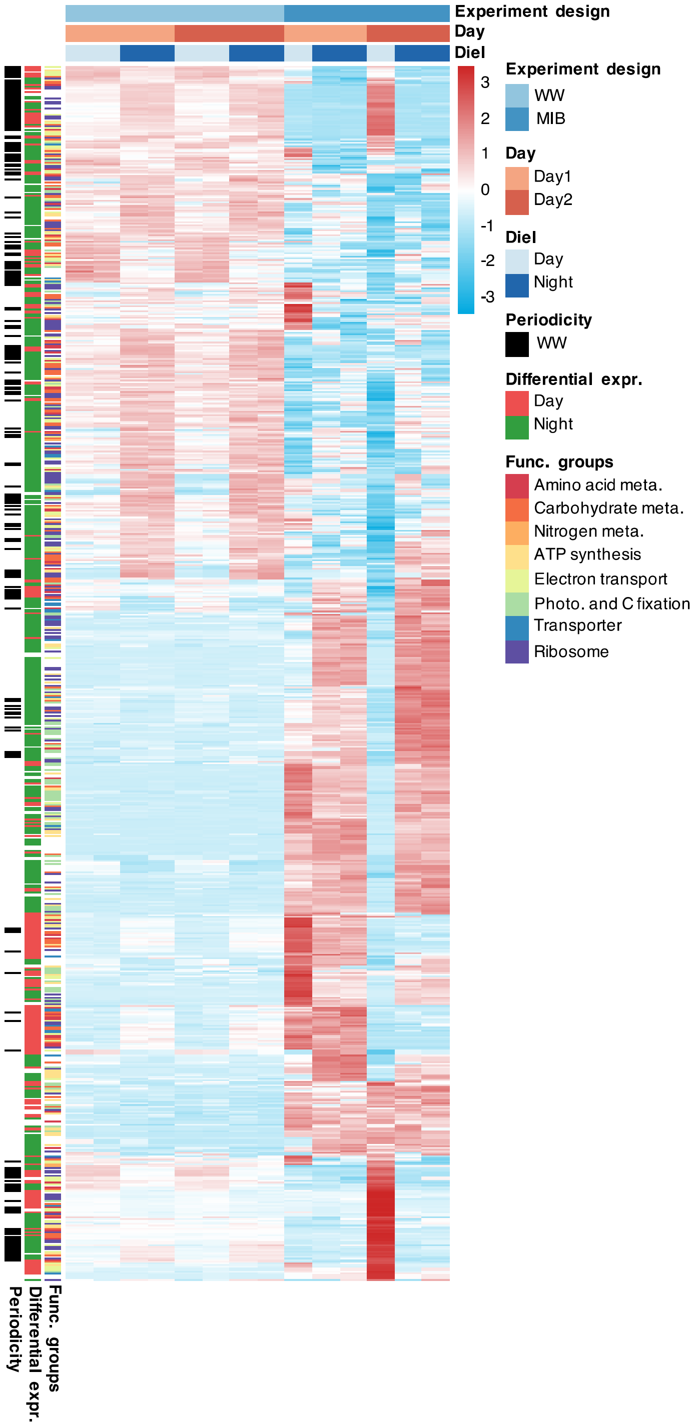

Figure S5. The expression pattern of microbiome genes between the microbiome (MIB) and whole water (WW) samples.

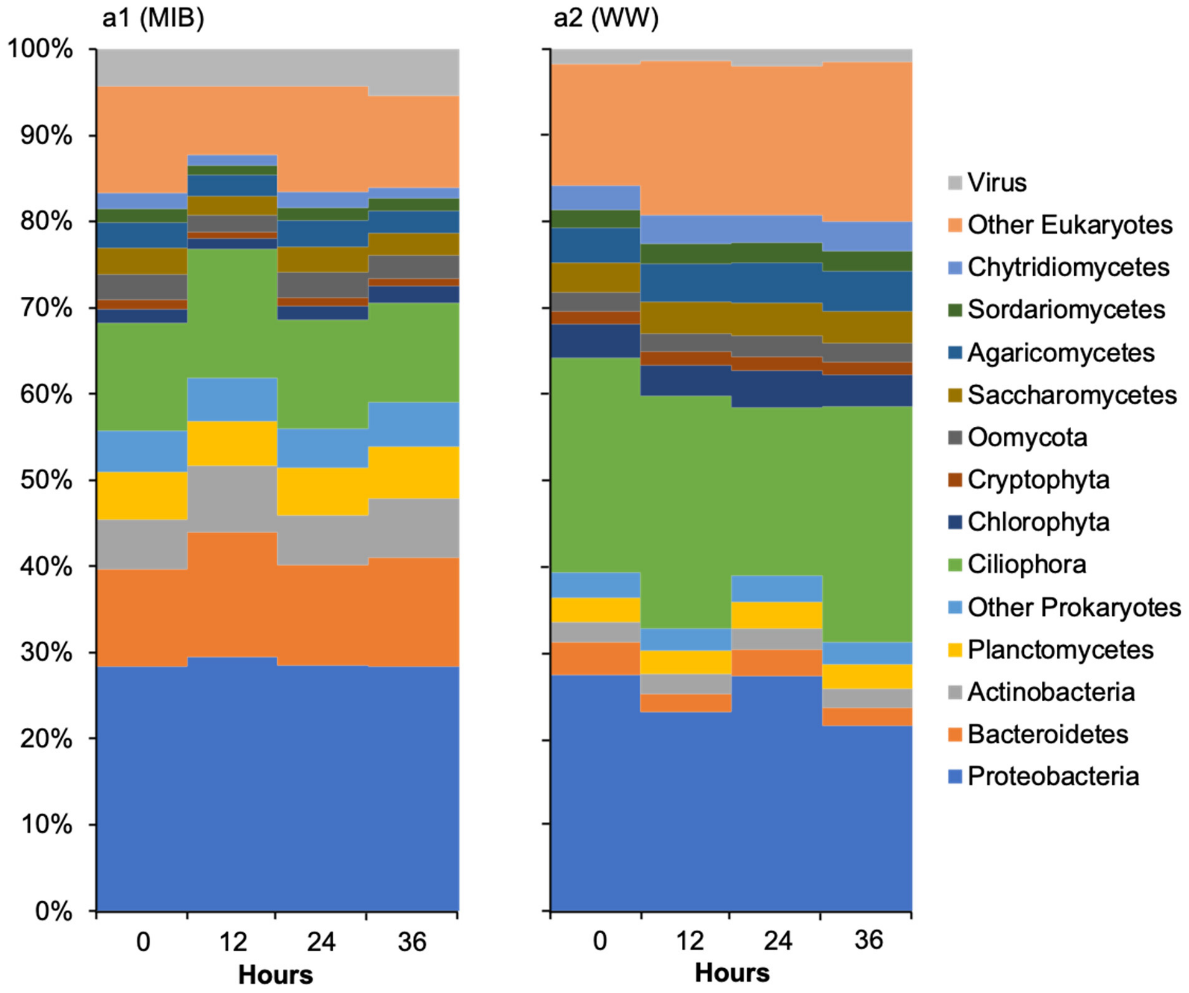

Figure S6 Microbiome communities identified in the MIBs and WWs. Only those taxa with relative abundance > 0.1% were shown.
